# Targeting Ero1L by Parthenolide Alleviates Cellular Senescence and Fibrosis of Localized Scleroderma by Regulating Mitochondria‐Associated Endoplasmic Reticulum Membranes Stabilization

**DOI:** 10.1111/acel.70738

**Published:** 2026-09-29

**Authors:** Fei Wang, Ru Xing, Meijuan Xie, Yimin Qin, Yunchao Zhang, Dan Cao, Gu He, Xiaoyan Lyu

**Affiliations:** ^1^ Department of Dermatology West China Hospital, Sichuan University Chengdu China; ^2^ Laboratory of Dermatology, Clinical Institute of Inflammation and Immunology, Frontiers Science Center for Disease‐Related Molecular Network, West China Hospital Sichuan University Chengdu China; ^3^ Department of Dermatology Lanzhou University Second Hospital Lanzhou China; ^4^ The Second Clinical Medical School Lanzhou University Lanzhou China; ^5^ School of Basic Medical Sciences Harbin Medical University Harbin China

**Keywords:** cellular senescence, Ero1L, fibrosis, localized scleroderma, mitochondria associated‐endoplasmic reticulum membrane, parthenolide

## Abstract

Mitochondrial dysfunction drives scleroderma pathogenesis. The mitochondria‐associated endoplasmic reticulum membrane (MAM) regulates mitochondrial function. Parthenolide (PTL) has protective effects on mitochondria. However, its specific role and mechanisms in localized scleroderma (LoS) remain unclear. This study investigates whether PTL reduces senescence and skin fibrosis by stabilizing MAM in LoS and examines the protective effects of a synthesized thermosensitive controlled‐release hydrogel (F127@PLGA@PTL) against LoS. We employed transmission electron microscopy (TEM) to observe MAM abnormalities in LoS skin lesions. Other multiple molecular biology experiments were conducted to investigate how PTL protects against LoS in TGF‐β‐induced human dermal fibroblasts (HDFs) and a murine model of LoS. RNA sequencing, molecular docking‐based inverse virtual screening, and surface plasmon resonance (SPR) were used to find possible targets of PTL. Emulsification‐volatilization was used to prepare the PTL‐loaded PLGA nanoparticles. MAM's calcium ion transport function is enhanced, and its quantity increases in LoS. PTL can ameliorate senescence and fibrosis in TGF‐β‐induced HDF cells and the bleomycin‐induced LoS model by regulating the IP3R1/Grp75/VDAC1 complex and repairing mitochondrial damage. Notably, mechanistically, this protective effect was mediated through Ero1L suppression, with ARG‐449 identified as the critical residue for PTL–Ero1L binding. Ero1L overexpression reversed the protective effects of PTL. Additionally, Ero1L knockdown also exhibits anti‐senescence and antifibrotic effects on TGF‐β‐induced HDFs. Furthermore, F127@PLGA@PTL demonstrates excellent sustained‐release properties and significant therapeutic efficacy in the mouse model of LoS. The study demonstrates that PTL alleviates fibrosis of LoS by regulating Ero1L‐mediated MAM stabilization.

## Graphical abstract

PTL inhibits cell senescence and alleviates fibrosis of Los by regulating Ero1L‐mediated mitochondria‐associated endoplasmic reticulum membranes stabilization.

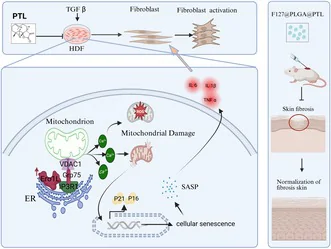

**Abbreviations**
ANOVA: analy‐sis of variance analysis
AOD: average optical density
Ca^2+^
: calcium ions
COL1A1: type I collagen
DCM: dichloromethane
DEGs: differential expression of genes
DMSO: dimethyl sulfoxide
ER: endoplasmic reticulum
HDFs: human dermal fibroblasts
HPLC: high‐performance liquid chromatography
LoS: localized scleroderma
MAM: mitochondria associated‐endoplasmic reticulum membrane
MMP: mitochondrial membrane potential
mRSS: modified Rodnan skin score
mtROS: mitochondrial reactive oxygen species
PBS: phosphate‐buffered saline
PTL: parthenolide
PVA: polyvinyl alcohol
RNA sequencing: RNA‐Seq
ROS: reactive oxygen species
SASP: senescence‐associated secretory phenotype
SEM: scanning electron microscopy
TEM: transmission electron microscope
TFAM: Mitochondrial Transcription Factor A
α‐SMA: α‐smooth muscle actin

## 1 Introduction

Localized scleroderma (LoS) is a rare chronic connective tissue disease characterized by skin induration and fibrosis within the dermal or subcutaneous layers. In severe cases, morphea may be accompanied by skin ulcers and disfiguring cutaneous lesions. As the disease progresses, it can result in joint deformity, significant restriction of joint movement (Defabianis 2003), and an increased the risk of future fractures (Barrera Vigo et al. 2008). It creates a substantial mental and physical burden for the patient and simultaneously elevates expenses. To date, treatment for LoS primarily relies on immunosuppressants and glucocorticoids (Constantin et al. 2018; Zulian et al. 2019). Although new treatment modalities such as tocilizumab and JAK inhibitors are emerging (LaChance et al. 2022), no available therapy can reverse skin fibrosis of LoS (Wang et al. 2022).

Globally, there is no effective systematic treatment available to cure LoS. Hence, it is essential to have a deep understanding of the molecular mechanisms underlying LoS and to find effective therapeutic drugs. Previously, the pathogenesis of scleroderma involves inflammation, fibrosis and microvascular injury. Furthermore, mitochondrial dysfunction contributes to the pathogenesis of scleroderma. Fibroblast activation is a common pathological feature of both LoS and SSc, mitochondrial damage is a core factor driving fibroblast activation (Cantanhede et al. 2022). Mitochondria are the main source of reactive oxygen species (ROS), and oxidative stress is a consequence of mitochondrial dysfunction (Hasberg et al. 2026). Oxidative stress plays an important role in the pathogenesis of LoS (Akbaş et al. 2024; Kilinc et al. 2016), therefore, mitochondrial damage may be involved in the pathogenesis of LoS. Dysfunctional mitochondrial calcium homeostasis is a risk factor for inducing mtROS accumulation. The mitochondria‐associated endoplasmic reticulum membrane (MAM) serves as a critical structure for mitochondrial Ca^2+^ generation (Padhiar et al. 2025). IP3R1, GRP75, and VDAC1 form the Ca^2+^ channel transport complex located on the MAM (Thoudam et al. 2019). Ca^2+^ influx into mitochondria is linked to mitochondrial dysfunction, elevated production of mitochondrial reactive oxygen species (ROS), and cellular senescence (Satrústegui et al. 1996). Recent research has demonstrated that cellular senescence plays a role in the pathogenesis of scleroderma (Bueno et al. 2020). Senescent cells contribute to chronic inflammation and epithelial‐mesenchymal transition, promoting fibroblasts and macrophages to adopt a fibrotic phenotype. This process ultimately leads to fibrosis through the secretion of the senescence‐associated secretory phenotype (SASP) (Liu et al. 2022; Tsou et al. 2022). Hence, the mitochondrial calcium influx regulated by MAM could serve as a potential target for addressing LoS pathogenesis.

Parthenolide (PTL), a sesquiterpene lactone derived from the feverfew plant, exhibits multiple biological activities, including anti‐inflammatory, antioxidative, pro‐apoptotic, and antitumor effects (Shariat Razavi et al. 2024; Wang et al. 2025; Zhang et al. 2025). Semisynthetic modification of PTL enables it to reduce MMP and decrease mtROS, thereby rescuing mitochondrial function (Lei et al. 2020). Mitochondrial dysfunction has been reported in scleroderma and is known to induce myofibroblast activation (Zhou et al. 2022). Thus, improving mitochondrial function can be a strategy to alleviate skin fibrosis in scleroderma patients. Given that PTL can improve mitochondrial function, we speculated that PTL may help to alleviate scleroderma. PTL shows anti‐fibrotic effects in many fibrotic diseases (Cui et al. 2021; Li et al. 2018; Zhang et al. 2022), fibroblast activation is the common pathogenesis of fibrotic disorders. However, limited data are available regarding the influence of PTL on fibroblast activation in skin fibrosis. This is an important research gap, so we attempted to address this gap by conducting this research. Although PTL demonstrates significant antioxidant activity and provides protection against mitochondrial dysfunction, Ca^2+^ overload is a significant contributor to mitochondrial dysfunction (Li et al. 2021). However, no literature has documented how PTL modulates the assembly of MAMs or regulates ER‐mitochondrial Ca^2+^ flux. Regrettably, PTL's clinical application is hindered by its poor water solubility and low bioavailability. PLGA nanoparticles can effectively overcome PTL's solubility limitations, while F127 thermoresponsive hydrogels have a sustained‐release effect. Consequently, the combination of F127 and PLGA presents a viable strategy for PTL delivery.

This study employed a BLM‐induced LoS mice model to investigate the anti‐senescence and anti‐fibrotic efficacy of PTL on skin fibrosis. Additionally, the underlying mechanisms of PTL's anti‐senescence and anti‐fibrotic actions were analyzed by RNA sequencing (RNA‐Seq) Co‐immunoprecipitation, transmission electron microscopy (TEM) and molecular docking. We found that PTL alleviates the skin fibrosis by regulating MAM homeostasis and calcium flux at MAM sites through targeting Ero1L. Finally, we found that F127@PLGA@PTL exhibited anti‐aging and anti‐fibrotic effects in a Los mouse model. In this study, we elucidated anti‐senescence and anti‐fibrotic efficacy of PTL and provided mechanistic insights into these effects. Meanwhile, we improved the delivery of PTL, offering greater potential for its clinical application.

## 2 Materials and Methods

### 2.1 Skin Tissue Collection

Skin tissue samples from 30 morphea patients treated at West China Hospital of Sichuan University between February 2024 and August 2024 were collected for immunohistochemical analysis. Which was approved by the clinical ethics committee in West China Hospital, Sichuan University. All patients provided their informed consent in writing. Adjacent normal skin tissues were collected from 15 patients with melanocytic nevi as controls. Healthy controls were selected to match the LoS group in age and gender (Table S1). Additionally, skin tissue samples were collected from three morphea patients treated at West China Hospital between August 2024 and October 2024 for TEM analysis, with perilesional skin from three pigmented nevus patients serving as healthy controls.

### 2.2 Immunohistochemistry and TEM

For immunohistochemistry, the skin biopsy was fixed in 3.7% paraformaldehyde, processed, and embedded in paraffin. After antigen retrieval and blocking, slides were incubated with primary and secondary antibodies, followed by chromogenic visualization. Three random fields at 400× magnification were captured using a light microscope, and staining intensity and area were analyzed with Image‐Pro Plus software. Protein expression was quantified using the average optical density (AOD) of each field. For TEM, ultrathin sections were prepared from tissue fixed in 3% glutaraldehyde and 1% osmium tetroxide, then observed using a TEM to measure the distance between mitochondria and the ER.

### 2.3 HDFs Culture

HDFs were obtained from the Department of Dermatology at West China Hospital, Sichuan University. The HDFs were cultured in 25 cm^2^ flasks (Nest, Wuxi, China) using complete medium containing 10% fetal bovine serum (FBS; ExCell, Suzhou, China) and 1% penicillin–streptomycin (Beyotime, Shanghai, China).

### 2.4 TGF‐β Induces Fibrosis in HDFs

TGF‐β triggers fibrosis in HDFs. HDFs were stimulated with TGF‐β (10 ng/mL) in complete DMEM F12 medium and cultured in a 37°C, 5% CO_2_ incubator for 48 h.

### 2.5 CCK8 Was Used to Determine the Optimal Time and Concentration of PTL

HDFs cells were exposed to PTL at varying concentrations (2.5, 5, 10, 20 μM) over a period of 24 h. Cell viability was assessed by the CCK8 assay. Cell survival in 24, 48, and 72 h was detected by the CCK8 assay to investigate the optimal time points and concentrations of PTL.

### 2.6 Real‐Time Quantitative Polymerase Chain Reaction (RT‐qPCR)

The total RNA was isolated using a total RNA isolation kit (Aidlab, Beijing, China); cDNA was synthesized using a cDNA reverse transcription kit (Qin Ke, HuBei, China). Real‐time PCR was conducted using SYBR Green PCR master mix on a Q6 Real‐Time PCR System, and relative expression levels were calculated with the 2^−ΔΔCT^ method. Primer sequences are listed in Supporting Information S1: Table S2.

### 2.7 Western Blot

RIPA lysis buffer was used to lyse the cells, and supernatant was gathered. Solubilized protein extracts were loaded into each lane, separated by SDS‐PAGE, transferred onto PVDF membranes, and subsequently exposed to the relevant primary antibody. The analysis of the results was conducted using ImageJ software. Antibody information is provided as Supporting Information (Table S3).

### 2.8 Co‐Immunoprecipitation

The cells were lysed with IP lysis buffer. To remove nonspecific binding, 1 mg of protein was incubated with 50 μL of Protein A/G Agarose beads. The correct amounts of GRP75 (2 μg) antibody and non‐specific IgG were then added and incubated overnight at 4°C. The precipitate was then collected by centrifugation for further Western blot analysis.

### 2.9 SA‐β‐Gal Staining

A Senescence β‐Galactosidase Staining Kit (Beyotime, China) was used to perform SA‐β‐Gal staining. The level of staining was examined and images were captured using a standard optical microscope. Senescent cells appear blue.

### 2.10 EdU Assay

Cell proliferation was evaluated using the BeyoClick EdU‐488 kit (Beyotime) according to the manufacturer's protocol. Cells were incubated with 10 μM EdU for 2 h, fixed with immunostaining fixative P0098, and permeabilized with immunostaining strong permeabilization solution P0097. The click reaction was performed to detect EdU incorporation, and nuclei were counterstained with Hoechst 33342. EdU‐positive cells were visualized by inverted epifluorescence microscope (Leica, DMi8).

### 2.11 Confocal Laser Scanning Microscope

The cells were treated with Mito‐Green working solution (100 nM) and then incubated in the dark at 37°C for 30 min. After incubation, the cells were washed three times for 3 min each with 1X PBS, and then incubated with the ER‐Tracker Red working solution (1 μmol/L) at 37°C in the dark for 30 min. Next, 1 mL of cell culture medium was added, and the cells were observed under a laser confocal microscope.

### 2.12 Flow Cytometry Quantified Both the MMP, Mitochondrial Ca^2+^ and the Production of ROS

MMP, ROS, and mitochondrial Ca^2+^ were detected by flow cytometry analysis. The MMP was measured using the JC‐1 MMP Assay Kit (Biyuntian Biotechnology, Shanghai, China). Mitochondria‐mediated ROS generation was detected with the mitochondrial superoxide indicator MitoSOX Red (Invitrogen). The Ca^2+^ concentration levels in the mitochondria were detected using 3 μM rhod‐2 AM (Maokang, Shanghai, China).

### 2.13 RNA Sequencing

In total, nine cell samples (three from the Control group, three from the TGF‐β group, and three from the TGF‐β + PTL group) were subjected to high‐throughput RNA sequencing. Transcriptome RNA sequencing (RNA‐Seq) was performed using Illumina high‐throughput RNA sequencing.

### 2.14 Molecular Docking‐Based Inverse Virtual Screening

Homologous structures of 2500 gene‐encoded proteins were downloaded from the Protein Data Bank (PDB), and the ligand library comprised of drugs was obtained from the ZINC15 database. Inverse virtual screening was performed using AutoDock Vina. A set of 100 candidate reference genes was selected based on binding free energy. The intersection of Top100 and DEGs was considered a potential target gene of PTL.

### 2.15 Surface Plasmon Resonance (SPR) Assay

SPR‐based binding assays were performed on a Biacore 1K instrument (Cytiva, USA) to measure the interaction between PTL and Ero1L. Ero1L recombinant protein (Sino Biological, China) (50 μg/mL) was coupled to a CM5 chip. After blocking with ethanolamine, B4 was diluted to varying concentrations and injected at a flow rate of 10 μL/min, with a 100‐s association phase followed by a 120‐s dissociation phase. Binding data were collected and analyzed using the Biacore 1K evaluation software (GE Healthcare).

### 2.16 Single‐Cell RNA‐Sequencing

The single‐cell RNA‐seq dataset GSE264508 (including fourteen LoS patients and fourteen healthy controls) was analyzed. Cells were clustered using the Seurat R package (version 3.1.1), uniform manifold approximation and projection (UMAP) was used for cell cluster visualization. Violin plots were plotted using Seurat's VlnPlot() function.

### 2.17 siRNA Transfection

Cells were transfected with Ero1L siRNA (siEro1L) and negative control siRNA (siNC) for 48 h. Table S4 lists the siRNA sequences that were used in this study.

### 2.18 Ero1L Overexpression

The negative control lentivirus and Ero1L overexpression lentivirus were produced by Jima Com (Shanghai, China). The following day, virus stocks were serially diluted 10‐fold and then added to the cells. Fluorescent signals and WB analyses were performed 48 h after infection.

### 2.19 Preparation and Characteristic of F127@PLGA@ PTL

We accurately weighed 40 mg of PTL powder and completely dissolved it in 2 mL of dimethyl sulfoxide (DMSO). We then added 2 mL of dichloromethane (DCM) to the mixture, followed by the precise incorporation of 10 mg PLGA powder, with 10 min ultrasonication to ensure complete dissolution. Subsequently, we slowly dripped the resulting emulsion into a 0.5% polyvinyl alcohol (PVA) solution and transferred the system to a 2% isopropanol solution, stirring continuously at room temperature for 4 h to achieve solidification. Finally, we purified the PLGA@PTL nanoparticles, dispersed the final product in sterile phosphate‐buffered saline (PBS), and added F127 powder to adjust its final concentration to 25% (w/v), thereby completing the functionalization of the nanoformulation. We employed TEM to examine the internal microstructure of samples and scanning electron microscopy (SEM) to observe the surface morphology of hydrogel microspheres. A rotational rheometer was utilized to perform stress sweep, frequency sweep, and temperature sweep tests. High‐performance liquid chromatography (HPLC) was applied to monitor drug release profiles.

### 2.20 LoS Animal Models and Treatment Protocol

A mouse model of LoS was established by shaving the dorsal hair and administering daily subcutaneous bleomycin injections for 28 consecutive days in female C57BL/6 mice. The modeled mice were randomly divided into four groups for different treatments: negative control group (saline), single‐drug group (PTL, 20 mg/kg), vehicle control group (F127), and experimental treatment group (F127@PLGA@PTL, 70 mg/kg). F127 and F127@PLGA@PTL group received treatment once a week for 28 days, and the remaining groups were treated every other day to evaluate therapeutic efficacy. Mice were euthanized 4 weeks after the therapy, and the skin samples were collected from a 4 cm^2^ shaved area for analysis. All animal experiments followed ethical guidelines approved by the ethics committee of West China Hospital, Sichuan University (Approval no. 2024(1076)).

### 2.21 Histological Valuation

The mouse skin tissue was embedded in paraffin, followed by HE staining and Masson staining.

### 2.22 ELISA

We performed ELISA detection on cell supernatants or mouse serum. The ELISA assays were conducted using an Elabscience ELISA kit.

### 2.23 Statistical Analysis

All values were presented as mean and standard deviation. The results were analyzed by *t*‐test or one‐way analysis of variance analysis (ANOVA). Data are presented as mean ± SD, from three independent biological replicates. Probability values *p* < 0.05 were considered significant.

## 3 Results

### 3.1 PTL Inhibits TGF‐β‐Induced Cell Senescence and Fibrosis in HDFs

To identify the optimal concentration and effect time of PTL, a CCK‐8 assay was used. We applied different concentrations (2.5, 5, 10, 20, and 40 μM) of PTL to treat HDF cells. We observed a notable decrease in cell viability at concentrations of 10 μM (*p* < 0.05) (Figure S1A) after 24 h. For subsequent experiments, we determined that 5 μM was the ideal concentration. We assessed the influence of PTL on HDF cells with various exposure times (24, 48, and 72 h). The results indicated that 48 h was the ideal timing for intervention (Figure S1B). Activated HDF cells were treated with low, medium, and high concentrations (1.25, 2.5, and 5 μM) of PTL. Our results showed that anti‐fibrotic effects were observed only at high concentrations of parthenolide, whereas anti‐aging effects were evident at both high and medium concentrations (Figure S1C–F). These findings indicate that the anti‐aging and anti‐fibrotic effects of PTL are concentration‐dependent. The β‐galactosidase staining results demonstrated significantly higher β‐galactosidase expression in elderly subjects compared to pediatric populations (Figure 1A). β‐galactosidase, P16, IL‐6, IL‐1β, and TNF‐α expression in LoS patients was significantly increased (Figure 1A–H). PTL can reduce β‐galactosidase‐positive cells and increase EdU‐positive cells in TGF‐β1‐induced HDF (Figure 1I–K, Figure S2). Meanwhile, PTL significantly downregulated both mRNA and protein levels of p21 and p16 in TGF‐β1‐induced HDFs, while also reducing secreted levels of IL‐6, IL‐1β, and TNF‐α in the cell supernatant (Figure 1N–T). Of note, we observed that PTL treatment led to the downregulation of mRNA expression levels for both α‐smooth muscle actin (α‐SMA) and Type I collagen (COL1A1) (Figure 1U,Y). These results showed that treatment with PTL attenuates TGF‐β‐induced fibrosis. We found that D + Q treatment significantly reduced the expression levels of COL1A1 and SMA proteins in TGF‐β‐treated HDFs, demonstrating that senescent cell clearance attenuates TGF‐β1‐induced fibrosis.

**FIGURE 1:**
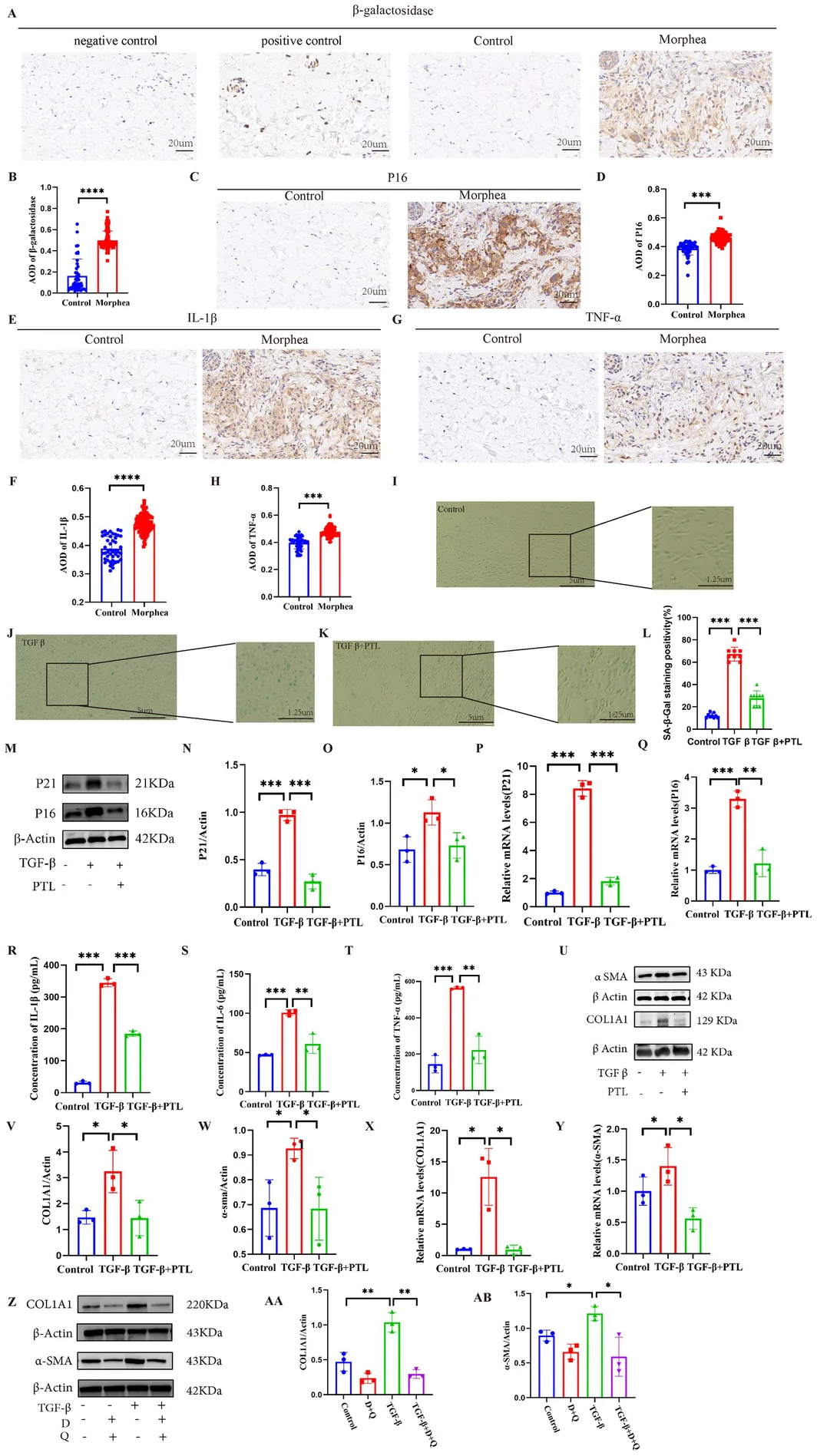
PTL inhibits TGF‐β‐induced cell senescence and fibrosis in human dermal fibroblasts (HDFs). (A) Immunohistochemistry for β‐galactosidase in human skin (positive staining appears brown. Control: *n* = 15, Morphea: *n* = 30 scale bar = 20 μm). (B) AOD levels of β‐galactosidase. (C) Immunohistochemistry for P16 in human skin (positive staining appears brown. Control: *n* = 15, Morphea: *n* = 30 scale bar = 20 μm). (D) AOD levels of P16. (E) Immunohistochemistry for IL‐1β in human skin (positive staining appears brown. Control: *n* = 15, Morphea: *n* = 30 scale bar = 20 μm). (F) AOD levels of IL‐1β. (G) Immunohistochemistry for TNF‐α in human skin (positive staining appears brown. Control: *n* = 15, Morphea: *n* = 30 scale bar = 20 μm). (H) AOD levels of TNF‐α. (I–K) β‐Galactosidase activity of HDF cells. (*n* = 3, scale bar = 5 μm) (L) Quantification of β‐galactosidase positive cells. (M–O) Western blot analysis was performed to assess the expression of P21 and P16 proteins in HDF cells from each group; Semi‐quantitative analysis was conducted with normalization to β‐Actin as the internal control (*n* = 3). (P, Q) RT‐PCR to examine P21 and P16 mRNA expression (*n* = 3). (R–T) The concentration of TNF α, IL‐1β and IL‐6 in cell supernatants was tested by ELISA (*n* = 3). (U–W). Western blot analysis was performed to assess the expression of COL1A1and α SMA proteins in HDF cells from each group; Semi‐quantitative analysis was conducted with normalization to β Actin as the internal control (*n* = 3). (X–W) RT‐PCR to examine COL1A1and α‐SMA mRNA expression. *p < 0.05, **p < 0.01, and ***p < 0.001 indicating statistical significance. (Z–AB) Western blot analysis was performed to assess the expression of COL1A1and α‐SMA proteins in HDF cells from each group (*n* = 3). Each experimental procedure was biologically replicated three times, each yielding similar results.

### 3.2 PTL Treatment Modulates the Homeostasis of MAMs, Thereby Improving Mitochondrial Function

The spatial proximity between the ER and mitochondria serves as an indicator of contact sites between these organelles. This proximity was significantly decreased in LoS patients, as shown in Figure 2A–C. PTL treatment can diminish the colocalization between mitochondria and the ER, thereby increasing the inter‐organelle distance (Figure 2D–G). Mitochondria dysfunction is an important trigger for cell senescence. ROS accumulation and MMP disruption are recognized as indicators of mitochondrial damage. PTL administration resulted in an increase in MMP and a decrease in ROS levels (Figure 2H–K), indicating a reduction in mitochondrial damage. The MAMs function as an important platform for the regulation of mitochondrial function.

**FIGURE 2:**
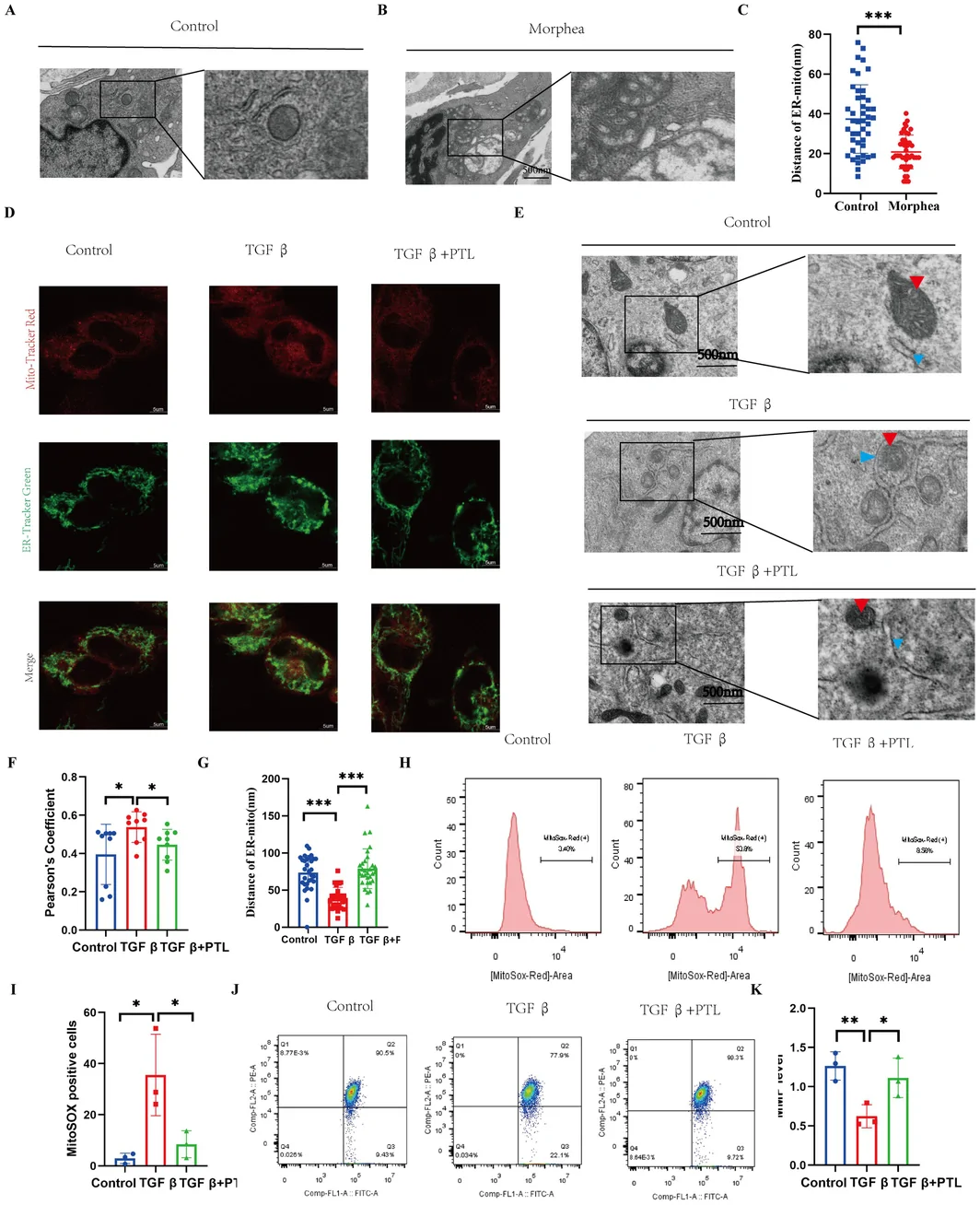
PTL can regulate homeostasis of MAM and mitochondrial damage. (A, B) TEM was used to monitor ultrastructural changes of the mitochondrion and endoplasmic reticulum in skin tissues. (Scale bar = 500 nm, *n* = 3) (C) Quantitative analysis of the distance between ER and mitochondria. (D, F) Laser confocal detection the colocalization of mitochondria and ER in vitro. (Scale bar = 5 μm, *n* = 3). (E) TEM was used to monitor ultrastructural changes of the mitochondrion and endoplasmic reticulum in HDF cells. (Scale bar = 5 μm, *n* = 3, The blue arrow represents mitochondria; the red arrow represents endoplasmic reticulum) (G) Quantitative analysis of the distance between ER and mitochondria. (H) ROS production in HDF cells was assessed using the BD LSR Fortessa Analyzer conducted with flow cytometry (*n* = 3). (I) Quantification of ROS levels. (J) The mitochondrial membrane potential (MMP) was tested by the JC‐1 assay to evaluate the mitochondrial activity (*n* = 3). (K) Quantification of MMP levels, *p < 0.05, **p < 0.01, and ***p < 0.001 indicating statistical significance. Each experimental procedure was biologically replicated three times, each yielding similar results.

### 3.3 PTL Treatment Downregulates Ero1L

To further explore the regulatory mechanism of PTL modulates the homeostasis of MAMs, we performed RNA sequencing. We identified 1111 differentially expressed genes (DEGs) induced by TGF β, and 290 DEGs following PTL treatment, using a threshold of |log (fold change, FC)| ≥ 1 and *p* < 0.05. Among them, 161 were common DEGs (Co‐DEGs) (Figure 3A–C). GO and KEGG pathway enrichment analyses showed that wound healing, collagen‐containing extracellular matrix, and ER lumen pathways were significantly enriched (Figure 3D,E). Through reverse virtual screening of 2600 genes, we identified the top 100 genes ranked by binding energy (Table S5). We took the intersection of top 100 genes and Co‐DEGs, and two genes (Ero1L and PI16) were identified (Figure 3F). The significant difference of PI16 after PTL intervention was less pronounced compared to that of Ero1L in the sequencing data (Figure 3G,H). In addition, compared to PI16, PTL exhibited lower binding energy with Ero1L (Table S5), which led us to prioritize Ero1L for subsequent analyses. Molecular docking indicated that PTL may bind to Ero1L with a binding energy of −7.3 kcal mol^−1^ (Figure 3I), which was further validated by SPR assays confirming a direct PTL‐Ero1L interaction (Figure 3J). Key binding residues, ARG‐449 and PHE‐62 of Ero1L, were identified through molecular docking simulations and theoretical bond analysis. Upon mutagenesis of these residues to alanine (ARG‐449A and PHE‐62A), we observed that the ARG‐449A mutation abrogated PTL's binding affinity for Ero1L (Figure 3L), whereas the PHE‐62A substitution exhibited no significant effect on the PTL‐Ero1L interaction (Figure 3K), confirming the specificity of the interaction. Similarly, PTL decreased Ero1L mRNA and protein expression in the TGF β‐treated cells (Figure 3N,O). UMAP analysis of all cells from 14 total samples of LS, with a total of 32,154 cells, clustered them into 15 main cell types (Figure 3P), Ero1L is expressed at greater level in activated fibroblasts compared to in other cell subtypes (Figure 3Q).

**FIGURE 3:**
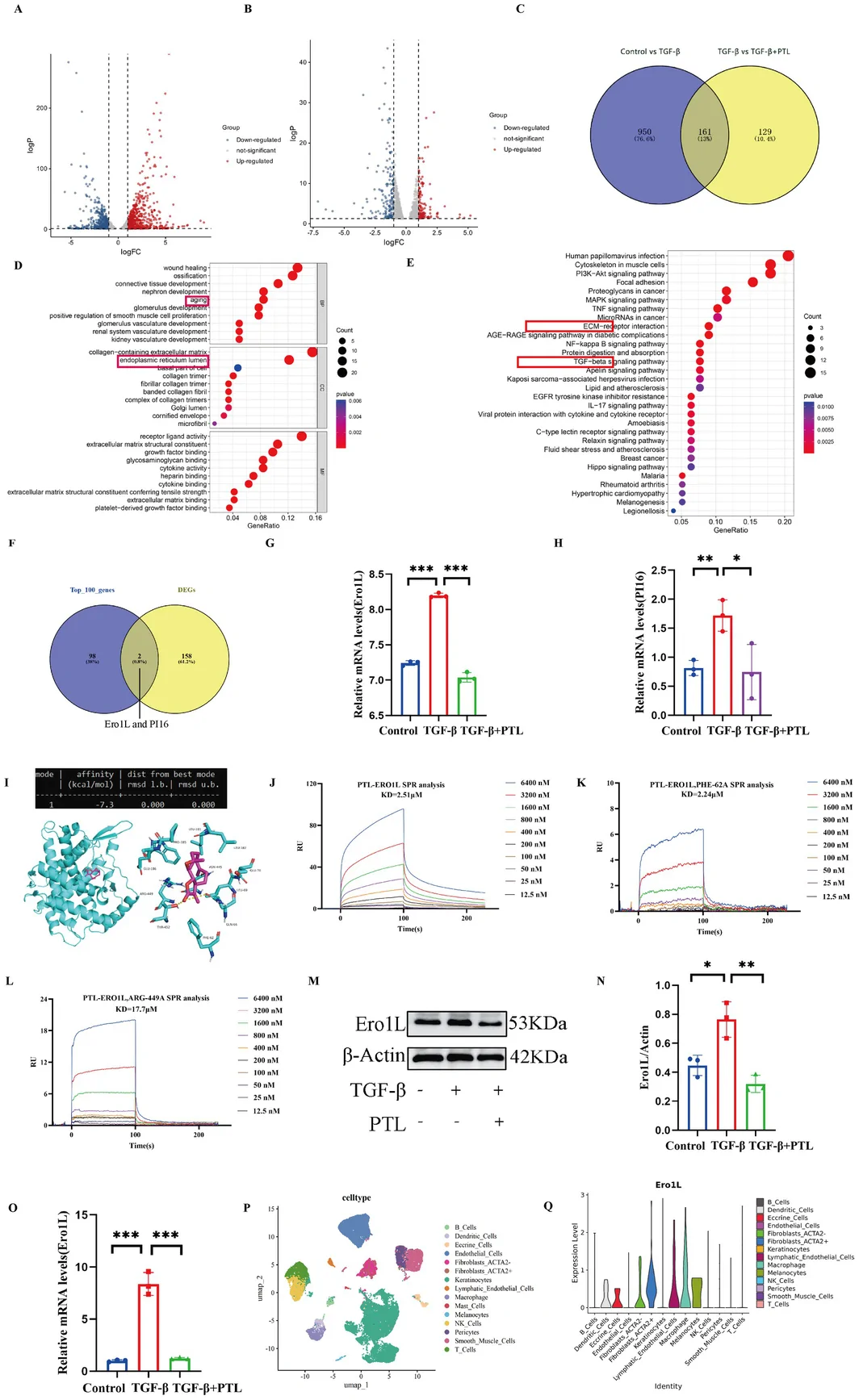
Reverse virtual screening, RNA sequencing and SPR reveals the PTL tendency to regulate MAM‐related gene Ero1L. (A) Volcano plot showing differentially expressed genes between control and TGF‐β + PTL (Control: *n* = 3, TGF‐β: *n* = 3, TGF‐β + PTL: *n* = 3) (B) Volcano plot showing differentially expressed genes between TGF‐β and TGF‐β + PTL. (C) Venn diagram showing co‐expressed differential genes (Co‐DEGs). (D, E) GO and KEGG analysis of Co‐DEGs. (F) The intersection of MAM‐related genes in LoS and Co‐DEGs was taken. (G) The levels of Ero1L expression in all groups in the sequencing data. (H) The levels of PI16 expression in all groups in the sequencing data. (I) Molecular docking simulation. (J) the binding sensorgram (for the interactions between PTL and Ero1L); (K) the binding sensorgram (for the interactions between PTL and PHE‐62A). (L) the binding sensorgram (for the interactions between PTL and ARG‐449A). (M, N) Western blot analysis was performed to assess the expression of Ero1L proteins in HDF cells from each group; semi‐quantitative analysis was conducted with normalization to β Actin as the internal control (*n* = 3). (O) RT‐PCR to examine Ero1L mRNA expression (*n* = 3). *p < 0.05, **p < 0.01, and ***p < 0.001 indicating statistical significance. (P) UMAP of all cells from 14 total samples of LS with a total of 32,154 cells clustered into 15 main cell types. (Q) The violin plots of Ero1L expression in different cell clusters. Each experimental procedure was biologically replicated three times, each yielding similar results.

### 3.4 PTL Normalizes the Ca^2+^ Transfer in MAM Region

GSEA enrichment analysis shows that Ero1L is enriched in organelle fusion and regulation of Ca^2+^ transport signaling pathways (Figure S3). IP3R, GRP75, and VDAC1 are classical calcium channel transporters located on the MAM. PTL down‐regulated both the mRNA and protein levels of IP3R1, GRP75, and VDAC1 in TGF‐β1 induced HDFs (Figure 4A–G). Data from co‐immunoprecipitation revealed a high interaction level between IP3R1 and Grp75, as well as between GRP75 and VDAC1 under TGF‐β1‐induced conditions (Figure 4H), which decreased after PTL treatment. Flow cytometry shows PTL can ameliorate mitochondrial calcium overload (Figure 4I–L).

**FIGURE 4:**
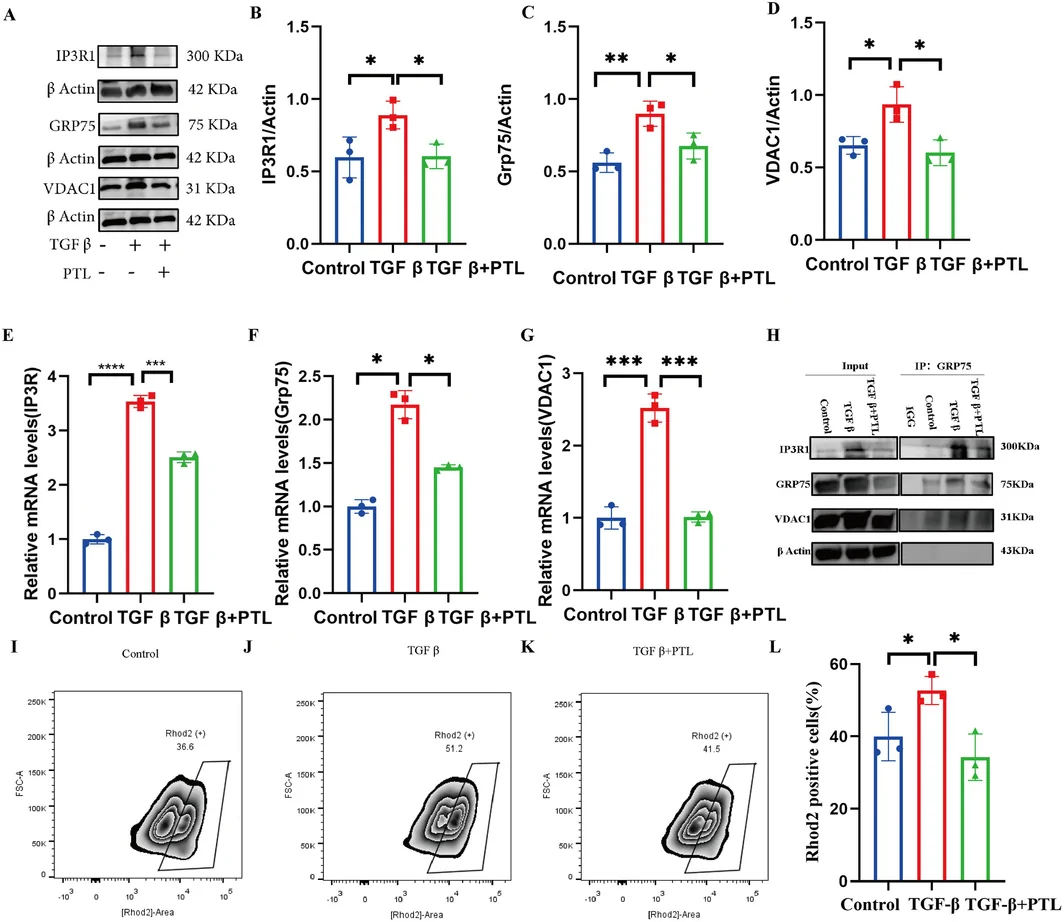
PTL decreased mitochondrial calcium, interaction level between IP3R, GRP75, and VDAC1 even with the presence of PTL. (A–D) Western blot analysis was performed to assess the expression of IP3R, GRP75 and VDAC1 proteins in HDF cells from each group; semi‐quantitative analysis was conducted with normalization to β‐actin as the internal control (*n* = 3). (E–G) RT‐PCR to examine IP3R, GRP75, and VDAC1 mRNA expression (*n* = 3). (H) Co‐immunoprecipitation (Co‐IP) assay showing the interaction between IP3R, GRP75 and VDAC1 (*n* = 3). (I, K) Mitochondrial calcium ion detection was detected by flow cytometry (*n* = 3). (L) Quantification of mitochondrial calcium ion levels.*p < 0.05, **p < 0.01, and ***p < 0.001 indicating statistical significance. Each experimental procedure was independently replicated three times, each yielding similar results. Each experimental procedure was biologically replicated three times, each yielding similar results.

### 3.5 Ero1L Overexpression Exacerbated Senescence and Fibrosis in HDFs Even With the Presence of PTL

Compared to the control group, the Ero1L‐overexpressing group showed elevated levels of Ero1L mRNA and protein. The successful overexpression of Ero1L was further confirmed by immunofluorescence (Figure S4A–D). In comparison to the TGF‐β1 + OE‐Ctrl group, treatment with PTL significantly suppressed the expression of senescence‐ and fibrosis‐associated proteins. However, this suppression was reversed following Ero1L overexpression (Figure S4E–J), which notably increased the expression levels of these proteins. These results indicate that Ero1L overexpression can counteract the anti‐fibrotic and anti‐senescent effects of PTL.

### 3.6 Ero1L Overexpression Disrupts the Homeostasis of MAM and Ca^2+^ Transfer at the MAM, Even in the Presence of PTL

According to the flow cytometry assay, overexpression of Ero1L increases ROS levels and decreases MMP levels, these results indicate that overexpression of Ero1L weakens the protective benefits of PTL on mitochondria (Figure 5A–J). PTL reduces TGF‐β‐induced MAM formation in HDFs, thereby restoring MAM homeostasis. Interestingly, overexpression of Ero1L reverses these effects. We used confocal microscopy to observe the effects of PTL treatment or Ero1L overexpression on mitochondrial and ER contact. The results showed that Ero1L overexpression attenuated the suppressive effect of PTL on mitochondrial and ER colocalization (Figure 5K,L). PTL down‐regulated mitochondrial calcium and interaction level between IP3R1 and Grp75, as well as between GRP75 and VDAC1, in TGF‐β‐induced HDFs. Overexpression of Ero1L reverses these effects (Figure S5A–G).

**FIGURE 5:**
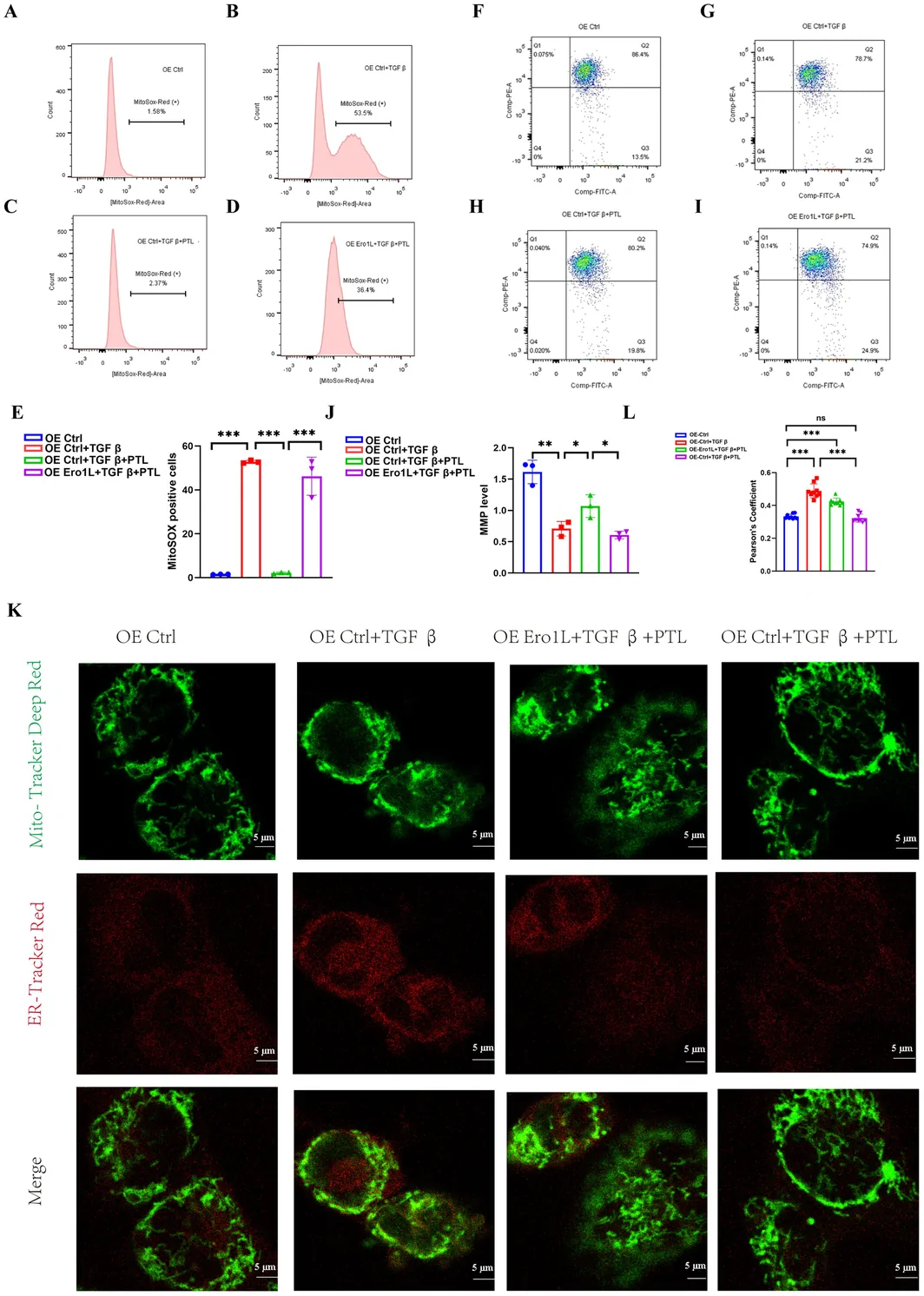
Ero1L overexpression exacerbates mitochondrial damage and disrupts the homeostasis of MAM even with the presence of PTL. (A–D) The mitoSOX red mitochondrial superoxide indicator was used to quantify mitochondrial ROS production (*n* = 3). (E) Quantification of mitochondrial ROS production. (F–I) The mitochondrial membrane potential (MMP) was tested by the JC‐1 assay to evaluate the mitochondrial activity (*n* = 3). (J) Quantification of MMP levels. (K) Laser confocal detection the colocalization of mitochondria and ER. (Scale bar = 5 μm, *n* = 3) (L) The quantification of the confocal immunofluorescence results.*p < 0.05, **p < 0.01, and ***p < 0.001 indicating statistical significance. Each experimental procedure was biologically replicated three times, each yielding similar results.

### 3.7 The Knockdown of Ero1L Ameliorated Fibrosis and Senescence, Restored MAM Homeostasis, and Alleviated Mitochondrial Calcium Overload

We further transfected HDF cells with SiEro1L to validate the role of Ero1L in LoS (Figure S6A–E). PCR and WB results indicated successful knockdown of Ero1L. We observed that the protein levels of both fibrosis‐related and senescence‐related markers were downregulated following Ero1L knockdown (Figure S6F–N). After Ero1L knockdown, mtROS levels were downregulated, while MMP levels were upregulated. These findings indicate that Ero1L exerts a protective effect against mitochondrial damage and can regulate the homeostasis of MAM (Figure S7A–J). Moreover, colocalization between mitochondria and the ER was reduced (Figure S7K,L). TEM imaging showed that Ero1L knockdown significantly increased the average distance between the ER and mitochondria (Figure S7M,N). Ero1L knockdown alleviated mitochondrial calcium overload. Furthermore, compared to the TGF‐β‐treated group, the binding interactions between IP3R and GRP75, as well as between GRP75 and VDAC1, were weaker in the SiEro1L + TGF‐β group (Figure S8A–J).

### 3.8 Characteristics of F127@PLGA@ PTL

To confirm the anti‐fibrotic effects of PTL in a mouse model of morphea, we developed a bleomycin‐induced model by administering daily subcutaneous injections of bleomycin. The 20 mg/kg dose demonstrated a greater anti‐fibrotic effect than the lower two doses (Figure S9). To enhance the water solubility of PTL, we designed a hydrogel delivery system that combines PLGA nanoparticles loaded with PTL and F127 thermo‐sensitive hydrogel. TEM revealed that PLGA@PTL exhibits a spherical morphology (Figure S10A). PLGA@PTL nanoparticles exhibit enhanced water solubility (Figure S10B). F127@PLGA@PTL exhibits injectability and thermoresponsive properties (Figure S10C–E). SEM images revealed a more compact three‐dimensional structure of the poloxamer hydrogel loaded with PLGA@PTL nanoparticles (Figure S10F). Figure S10G,H shows the XRD patterns of PLGA@PTL. F127@PLGA@PTL demonstrated a slower and more sustained release without an initial burst phase (Figure S10L). The rheological curve in Figure S9I displays F127 hydrogels display a temperature‐dependent rheological behavior. Frequency sweep rheology indicates that the incorporation of PLGA@PTL NPs has no significant effect on the rheological properties of the F127 hydrogel (Figure S100J). Strain sweep rheological analysis indicated that PLGA@PTL nanoparticles contribute to a certain enhancement in the network's resistance to deformation (Figure S10K). Good biocompatibility ensures the safety of biological tissues during operations. Live/dead staining and CCK8 assays further confirmed the good biocompatibility (Figure S10M,N).

### 3.9 F127@PLGA@PTL Alleviated Fibrosis and Senescence by Regulating MAMs Stabilization In Vivo

The assessment of dermal thickness, collagen content, and myofibroblast counts demonstrated that bleomycin successfully induced dermal fibrosis, while both PTL and F127@PLGA@PTL significantly attenuated skin fibrosis in murine models compared with the bleomycin‐induced group (Figure 6A–F). The study demonstrated that F127@PLGA@PTL significantly ameliorated collagen deposition and skin fibrosis (Figure 6U–W). Multiplex immunofluorescence analysis demonstrated that F127@PLGA@PTL significantly attenuated fibroblast activation, while exhibiting no measurable effects on vascular endothelial cells, keratinocytes, or Th2 lymphocytes (Figure 6G–L). F127@PLGA@PTL also reduced expression of Ero1L protein (Figure 6M,N). Additionally, F127@PLGA@PTL demonstrates anti‐aging effects (Figure 6O–T). Consistent with the in vitro results, PTL reduces mitochondria‐ER contact. F127@PLGA@PTL treatment also significantly reduced interaction levels between IP3R1 and Grp75, as well as between Grp75 and VDAC1 in BLM‐induced LoS (Figure S11A–E).

**FIGURE 6:**
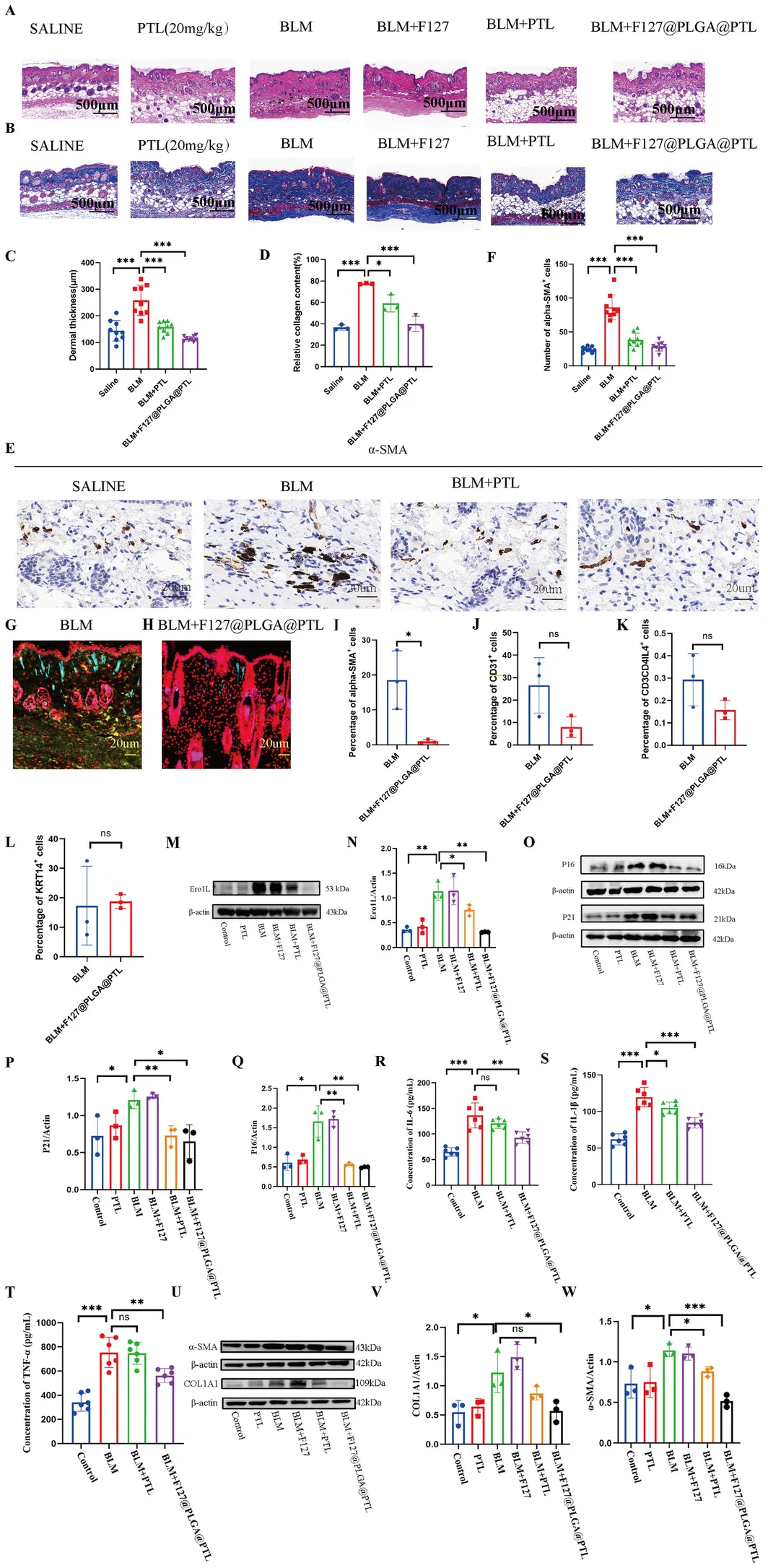
TPL@PLGA@F127 alleviated cell senescence and fibrosis in Vivo. (A) HE staining of skin in each group (*n* = 3). (B) Masson staining of skin in each group (*n* = 3). (C) Dermal thickness of skin (*n* = 3). (D) Relative collagen content (*n* = 3). (E) Representative images of alpha smooth muscle actin (α‐SMA) immunohistochemical analysis. (F) Number of α‐SMA‐positive cells in the dermis. (G, H) Multiplex immunofluorescence images of skin samples from LoS mice and F127@PLGA@PTL‐treated groups. Scale bar: 20 μm. IL‐4 (dark blue), DAPI (red), CD3 (green), CD4 (light yellow), α‐SMA (light blue), KRT14 (purple) and CD31 (dark yellow). (I–L) Quantification of cells (Activated fibroblasts; Vascular endothelial cells; Th2 cell population) in differentzones of LoS mouse and F127@PLGA@PTL‐treated skin tissues (*n* = 3 zones per tissue section). (M, N) Western blot analysis was performed to assess the expression of Ero1L proteins in each group; Semi‐quantitative analysis was conducted with normalization to β Actin as the internal control (*n* = 3). (O–Q) Western blot analysis was performed to assess the expression of P21 and P16 proteins in each group; Semi‐quantitative analysis was conducted with normalization to β Actin as the internal control (*n* = 3). (R–T) The expression of TNF‐α, IL‐1β and IL‐6 in cell supernatants was tested by ELISA (*n* = 3). (U–W). Western blot analysis was performed to assess the expression of COL1A1and α‐SMA proteins in each group; Semi‐quantitative analysis was conducted with normalization to β Actin as the internal control (*n* = 3). *p < 0.05, **p < 0.01, and ***p < 0.001 indicating statistical significance. Each experimental procedure was biologically replicated three times, each yielding similar results.

### 3.10 Ero1L Could Be a Potential Biomarker in LoS

We elaborated on the clinical significance of Ero1L. We discovered that Ero1L expression was significantly increased in skin biopsy samples from LoS patients (Figure S12A,B). Moreover, Ero1L levels show positive correlations both with cellular senescence and the modified Localized Scleroderma Skin Severity Index (mLoSSI) (Figure S12C,D).

## 4 Discussion

At present, the underlying mechanisms of LoS remain incompletely understood, and effective treatments are lacking. Mitochondrial damage is a major mechanism involved in the pathogenesis of scleroderma. Mitochondrial dysfunction leads to cellular aging. The regulation of mitochondrial function is predominantly mediated by the MAM and its associated Ca^2+^ channel complex. Nevertheless, it remains unclear whether there is homeostatic dysregulation of the MAM in scleroderma. This study provides preliminary evidence supporting the existence of abnormalities in the contacts between mitochondria and the ER in morphea.

PTL showed antifibrotic effects in liver, lung, and kidney, while also exhibiting mitochondrial protective effects; however, its role in scleroderma is less clear. This study has shown that PTL exhibits anti‐aging and anti‐fibrotic effects in scleroderma. It simultaneously helps regulate Ca^2+^ transport between the ER and mitochondria. Mechanistic studies showed that PTL regulates MAMs homeostasis and Ca^2+^ transport by targeting Ero1L, with key residue ARG‐449 mediating the PTL‐ Ero1L interaction.

In this study, we found that PTL exerts protective effects against cellular senescence and fibrosis in morphea. The anti‐fibrotic properties of PTL have been proven in several organs; our research further establishes its efficacy in mitigating skin fibrosis. Both LoS lesions and bleomycin‐induced skin fibrosis or TGF‐β1‐induced cellular fibrosis display elevated expression of senescence markers. Studies by Chiu et al. reported a significant upregulation of P16 and P21 in scleroderma dermal fibroblasts (Chiu et al. 2023), corroborating our findings. This study is the first to show the anti‐aging impact of PTL on mice experiencing skin fibrosis, indicating its promising potential for applications in the anti‐aging domain. This study suggested that PTL attenuates cellular senescence and fibrosis, however, the links between aging and fibrosis are not well understood in skin fibrosis. Whether PTL inhibits skin fibrosis by affecting cellular senescence is unclear. In this study, the clearance of senescent cells in each group using quercetin combined with dasatinib could alleviate fibroblast activation, suggesting that PTL alleviates fibrosis by modulating cellular senescence. In addition, the mechanism of aging is closely associated with mitochondrial dysfunction (Larsson 2010). In the morphea group, mitochondrial ridge abnormalities and breakage were observed. This study demonstrates that PTL alleviates mitochondrial damage by decreasing the production of mtROS and preventing the collapse of the mitochondrial membrane potential MMP (Ren et al. 2019). This findings align with our research results; however, the majority of studies have yielded results that are inconsistent with our outcomes. However, the majority of studies have reported results that contradict our observations. In the context of colorectal and ovarian cancers, PTL can induce mitochondrial dysfunction and trigger the mitochondrial pathway of apoptosis, thereby exerting therapeutic effects on tumors (Kim et al. 2012; Lee et al. 2012; LoBianco et al. 2022). Notably, PTL may exhibit contrasting roles across different diseases. This difference suggests that the biological effects of PTL are cell‐specific and regulated by the metabolic state of cells. In future work, more cell models should be employed to explore the function and underlying mechanism of PTL.

Although mitochondrial impairment is observed in scleroderma, the underlying mechanisms remain largely elusive. Irregular Ca^2+^ transport in the MAM area is closely associated with mitochondrial dysfunction (Filadi et al. 2017). Excessive Ca^2+^ accumulation in the mitochondria leads to damage, resulting in increased mitochondrial membrane permeability and a reduction in MMP (ΔΨm). This study found that abnormal MAM and calcium homeostasis were present in LoS, at the same time, we also observed mitochondrial calcium levels increased after TGF‐β stimulation. These results suggest that calcium signaling may be involved in the fibroblast activation. This phenomenon is consistent with other studies. In other studies, TGF‐β stimulation activates transient receptor potential vanilloid 4 (TRPV4) receptors, thereby promoting calcium ion influx into cells (Sharma et al. 2017). Actin polymerization induces the release of myocardin‐related transcription factor A (MRTF‐A) bound to actin monomers, which in turn leads to the translocation of nuclear MRTF‐A followed by initiation of α‐SMA gene transcription (Cen et al. 2003; Sandbo and Dulin 2011). TRPV and MCU Ion channels play a crucial role in the development of fibrosis. However, whether other calcium ion channels can influence fibroblast differentiation and function remains unclear. IP3R is an ER‐resident protein that forms a calcium transport complex with Grp75 and VDAC1, facilitating ER‐to‐mitochondria calcium flux to promote mitochondrial biogenesis and energy metabolism (Burkewitz et al. 2020). To date, no study has evaluated the relationship between IP3R and skin fibrosis till now. In this study, TGF‐β treatment upregulated both protein and mRNA expression of IP3R1, GRP75, and VDAC1 in HDFs, whereas PTL treatment significantly downregulated their expression. These results indicate that the IP3R1/GRP75/VDAC1 calcium channel complex is involved in the pathogenesis of scleroderma, and PTL can modulate IP3R1‐associated calcium signaling pathways.

Current evidence on MAM abnormalities is limited. Here, we found that PTL ameliorates fibrotic phenotypes by restoring MAM homeostasis and Ca^2+^ signaling. Although MAM abnormalities are documented in TGF‐β–treated lung fibroblasts (Ban et al. 2025), suggesting MAM disruption may be a shared feature across fibrotic diseases. Our TEM analysis revealed an increase in ER‐mitochondria contact sites in LoS lesions. Our study suggests that targeting MAM and calcium could be an effective therapeutic strategy for the treatment of skin fibrosis.

Cellular Ca^2+^ homeostasis is crucial for maintaining mitochondrial function; this highlights the necessity of investigating the mechanisms behind mitochondrial calcium ion regulation within cells in diseases associated with mitochondrial damage. The Ca^2+^ homeostasis of the MAM is regulated by a variety of genes. To investigate the regulatory mechanism of PTL on MAM in skin fibrosis, we performed RNA sequencing (RNA‐seq) and SPR analysis. These results indicated that ERO1L was upregulated in TGFβ1‐induced HDFs and was downregulated after PTL treatment. ERO1L, an oxidoreductase enzyme induced by hypoxic microenvironment response to ROS, is predominantly localized at the MAM (Guidarelli et al. 2023). Increased ero1L expression is recognized as a key indicator of ER stress. Studies have shown that scleroderma is associated with ER stress (Heindryckx et al. 2016; Lenna et al. 2013). Within the ER, the accumulation of unfolded proteins triggers an adaptive stress response known as the unfolded protein response (UPR). Ero1L is an enzyme primarily found in the ER involved in the protein folding process within the ER. Ero1L knockdown attenuates ER stress (Yeewa et al. 2024). One potential explanation for the elevated expression of Ero1L in scleroderma could be hypoxia is strongly associated with vascular damage and skin fibrosis of scleroderma. Patients with scleroderma often experience prolonged hypoxic conditions (He et al. 2022). Ero1L is very sensitive to hypoxia, resulting in increased expression levels in scleroderma. In our study, the knockdown of Ero1L attenuated ER stress and rescued mitochondrial dysfunction, corroborating the well‐established relationship between ER stress and mitochondrial damage documented in previous studies (Rainbolt et al. 2014; Raturi and Simmen 2013). ER stress not only impacts mitochondrial biogenesis but can also affects how mitochondria function, with elevated stress levels potentially exacerbating dysfunction. It has been demonstrated that Ero1L can modulate Ca^2+^ flux by regulating MCU channel activity (Anelli et al. 2012). However, the precise mechanism is not yet understood. Our results suggest that knockdown the ERO1L may reduce interactions among IP3R1‐GRP75‐VDAC1 complexes. The data here enhance our understanding of the mechanisms involved in its regulation of the Ca^2+^.

In recent years, more and more attention has been paid to the role of Ero1L in fibrotic diseases. Current studies have primarily focused on liver and pulmonary fibrosis (Druso et al. 2024; Fujii et al. 2017); however, no study has investigated the relationship between Ero1L and skin fibrosis. In this study, Ero1L knockdown could relieve fibroblast activation. Potential pro‐fibrotic mechanisms of Ero1L may involve its regulatory effect on the expression of COL1A1. Expression levels of COL1A1 were significantly elevated in fibrotic skin. Proteins in the ER can regulate the processing of COL1A1 (DiChiara et al. 2016). The folding of COL1A1 is closely related to the formation of disulfide bonds (DiChiara et al. 2018). Protein disulfide isomerase can facilitate the formation of correct disulfide bonds. Ero1L mediates the reoxidation of PDIA3 to maintain its enzymatic activity and drive fibrosis (Kumar et al. 2022). Therefore, Ero1L may indirectly promote fibrosis through redox regulation.

The role of Ero1L in cellular senescence has been reported as controversial. Inhibition of Ero1L can improve the physiological age, with pan‐neuronal knockdown of Ero1L significantly extending lifespan in Drosophila (Yeewa et al. 2024). Conversely, another study reported reduced ERO1L expression levels in aged monkeys compared to juveniles (Tan et al. 2020). This may be attributed to the function of a gene varying in different cell lines; understanding its relationship with age in different cells is an important future endeavor. Alterations in Ero1L expression are associated with the generation of ROS induced by UPR‐mediated apoptosis (Juan et al. 2021). Excessive ROS production can result in oxidative stress, causing damage to proteins, DNA, and lipids, and subsequently activating the p53, P21, and p16INK4A pathways, which lead to cell cycle arrest (Shamloo and Usluer 2019). Therefore, Ero1L can influence ROS production and thereby affect cellular outcomes.

PLGA nanoparticles serve as excellent drug delivery vehicles due to their demonstrated biocompatibility and controllable biodegradability. Furthermore, nanomedicines improve the dissolution of drugs with poor water solubility, which in turn can enhance their bioavailability (Guo et al. 2023). Hydrogels are three‐dimensional network structures that exhibit structural similarity to the biological tissues, representing one of the closest synthetic biomaterials to native tissue architecture (Vashist et al. 2014). The nanocomposite hydrogel, with NS particles embedded within its mesh network, exhibits significantly enhanced biological performance and mechanical toughness (Chen et al. 2018). The introduction of NS particles not only improves the mechanical strength of hydrogels, but also provides a controlled drug release profile. Based on this, the thermosensitive hydrogel Poloxamer F127 was selected as the matrix in this study, as it undergoes rapid sol–gel transition at body temperature, enabling localized drug retention and sustained release. Ultimately, we constructed a thermosensitive nanocomposite hydrogel delivery system (F127@PLGA@PTL), aiming to achieve dual sustained release of PTL. The CCK‐8 and Live/Dead staining tests proved that the hydrogels have excellent cell compatibility. F127@PLGA@PTL system could mitigate the initial burst release commonly observed with PLGA nanoparticles, resulting in a more sustained release profile. This property helps reduce dosing frequency and enhances therapeutic controllability.

PTL could reduce the expression of ERO1L, thereby regulating ER‐mitochondria contacts and Ca^2+^ transport into mitochondria from the ER. This study is the first to identify the role of ERO1L in the pathogenesis of LoS, suggesting it as a potential therapeutic target for PTL intervention. F127@PLGA@PTL demonstrated anti‐aging and anti‐fibrotic properties in this study. The integration of nanomedicines into hydrogels attenuates the initial burst release, leading to a smoother drug release profile. However, there are limitations to this study. The current findings from cell culture and mouse models necessitate further validation in human subjects.

## 5 Conclusion

Overall, this study represents the inaugural demonstration that PTL effectively alleviates senescence and skin fibrosis by regulating Ero1L‐mediated MAM stabilization. The findings confirm the therapeutic potential of PTL in treating senescence and skin fibrosis, while also offering new insights into its underlying molecular mechanism.

## Author Contributions

**Fei Wang:** data conceptualization, curation, writing – original draft preparation, methodology, software, visualization. **Xiaoyan Lyu:** investigation, methodology, software. **Gu He:** methodology, software. **Ru Xing:** writing – reviewing and editing. **Meijuan Xie:** visualization. **Yimin Qin:** software. **Yunchao Zhang:** software, validation. **Dan Cao:** writing – reviewing and editing.

## Funding

This work was supported by the Natural Science Foundation of Sichuan (2022NSFSC0713) and National Key R&D Program of China (2025YFA1804603).

## Ethics Statement

This study was approved by the ethics committee of West China Hospital of Sichuan University, and the ethical number are 2024 (1076) and 20250416009.

## Consent

The authors have nothing to report.

## Conflicts of Interest

The authors declare no conflicts of interest.

## Supporting information


**Figure S1:** The optimal concentration and optimal action time of PTL on HDFs. (A) Determine the optimal concentration of PTL. (B) Determine the optimal action time of PTL. (C‐F) Western blot analysis was performed to assess the expression of P21, α SMA, and COL1A1 proteins in HDF cells from each group; Semi‐quantitative analysis was conducted with normalization to β Actin as the internal control (*n* = 3).*p < 0.5, **p < 0.01 indicating statistical significance. Each experimental procedure was biologically replicated three times, each yielding similar results.
**Figure S2:** EdU assay determined the EdU positive rate. A Representative immunofluorescence images of EdU staining. B Quantification of EdU positive cells (*n* = 3), six visual fields were selected for counting in each picture. Percent EdU = EdU (+)/Hoechst 33342 (+) × 100. Each experimental procedure was biologically replicated three times, each yielding similar results.
**Figure S3:** GSEA analysis of Ero1L (Control vs. TGF β).
**Figure S4:** Overexpression of Ero1L inhibited the anti‐fibrotic and anti‐senescent effects of PTL. (A–D) Immunofluorescence, western blotting (WB) and qRT‐PCR results demonstrated that Ero1L is overexpressed in HDF cells (*n* = 3). (E–J) Western blot analysis was performed to assess the expression of proteins in HDF cells from each group; Semi‐quantitative analysis was conducted with normalization to β Actin as the internal control (*n* = 3). *p < 0.05, **p < 0.01, and ***p < 0.001 indicating statistical significance. Each experimental procedure was biologically replicated three times, each yielding similar results.
**Figure S5:** Overexpression of Ero1L increased mitochondrial calcium, interaction level between IP3R, GRP75 and VDAC1 even with the presence of PTL. (A, C) The calcium levels in the cells were measured by flow cytometry and quantification of calcium levels (*n* = 3). (B, D, E, F)Western blot analysis was performed to assess the expression of IP3R, GRP75 and VDAC1 proteins in HDF cells from each group; Semi‐quantitative analysis was conducted with normalization to β Actin as the internal control (*n* = 3). (G) Co‐immunoprecipitation (Co‐IP) assay showing the interaction between IP3R, GRP75 and VDAC1 (*n* = 3). *p < 0.05, **p < 0.01, and ***p < 0.001 indicating statistical significance. Each experimental procedure was biologically replicated three times, each yielding similar results.
**Figure S6:** Ero1L knockdown in HDF cells produced anti‐senescent and anti‐fibrotic effects. (A–C) Western blotting and PCR results showing ERO1L knockdown efficiency in HDF cells (*n* = 3). (D, E) Western blotting analysis confirmed successful knockdown of Ero1L in subsequent experiments; Semi‐quantitative analysis was conducted with normalization to β Actin as the internal control (*n* = 3). (F–K) Western blot analysis was performed to assess the expression of COL1A1, α SMA, P21 and P16 proteins in HDF cells from each group; Semi‐quantitative analysis was conducted with normalization to β Actin as the internal control (*n* = 3). (L–N) The expression of TNF α, IL‐1β and IL‐6 in cell supernatants was tested by ELISA.*p < 0.05, **p < 0.01, and ***p < 0.001 indicating statistical significance. Each experimental procedure was biologically replicated three times, each yielding similar results.
**Figure S7:** Ero1L knockdown helped to maintain MAM homeostasis and alleviate mitochondrial injury in HDF cells. (A–D, I) The mitoSOX red mitochondrial superoxide indicator was used to quantify mitochondrial ROS production. (*n* = 3) (E–H) The mitochondrial membrane potential (MMP) was tested by the JC‐1 assay to evaluate the mitochondrial activity. (*n* = 3) (J) Quantification of MMP levels. (K) The quantification of the confocal immunofluorescence results. (L) Laser confocal detection the colocalization of mitochondria and ER. (Scale bar = 5 μm, *n* = 3). (M) TEM was used to monitor ultrastructural changes of the mitochondrion and endoplasmic reticulum (Scale bar = 500 nm, *n* = 3, The blue arrow represents mitochondria; the red arrow represents endoplasmic reticulum). (N) Quantitative analysis of the distance between ER and mitochondria. (*p < 0.05, **p < 0.01, and ***p < 0.001 indicating statistical significance). Each experimental procedure was biologically replicated three times, each yielding similar results.
**Figure S8:** knockdown of Ero1L decreased mitochondrial calcium, interaction level between IP3R, GRP75 and VDAC1. (A–E) The calcium levels in the cells were measured by flow cytometry and quantification of calcium levels (*n* = 3). (F H I J)Western blot analysis was performed to assess the expression of IP3R, GRP75 and VDAC1 proteins in HDF cells from each group; Semi‐quantitative analysis was conducted with normalization to β Actin as the internal control (*n* = 3). (G) Co‐immunoprecipitation (Co‐IP) assay showing the interaction between IP3R, GRP75 and VDAC1. *p < 0.05 and **p < 0.01 indicating statistical significance. Each experimental procedure was biologically replicated three times, each yielding similar results.
**Figure S9:** HE and Masson trichrome staining. (A) HE staining. (B) Masson staining.
**Figure S10:** Characteristics of F127@PLGA@PTL. (A) Electron microscopy analysis (Scale bar = 100 nm, *n* = 3). (B) Water solubility of PTL, PLGA@PTL, and PTL plus PLGA. (C, D) Injectability properties of F127@PLGA@PTL. (E) Thermoresponsive properties of F127@PLGA@PTL. (F) SEM images of F127 and F127@PLGA@PTL. (G, H) XRD pattern of PTL and PLGA@PTL. (I–K) Temperature, frequency, and strain rheological curve figure of F127 and F127@PLGA@PTL. (L) Drug release profiles of PTL, PLGA@PTL, and F127@PLGA@PTL. (M) Live/dead cell staining illustrates viable (green) or dead (red) cells. (N) CCK8 assay showing cell viability in different groups and different time periods (*n* = 6 biological replicates).
**Figure S11:** TPL@PLGA@F127 could regulate calcium ion channel complex and MAM stabilization in vivo. (A–D)Western blot analysis was performed to assess the expression of IP3R, GRP75, and VDAC1 proteins in HDF cells from each group; Semi‐quantitative analysis was conducted with normalization to β Actin as the internal control (*n* = 3). (E) Co‐immunoprecipitation (Co‐IP) assay showing the interaction between IP3R, GRP75 and VDAC1. (F) TEM was used to monitor ultrastructural changes of the mitochondrion and endoplasmic reticulum (Scale bar = 500 nm, *n* = 3, The red arrow represents mitochondria; the blue arrow represents endoplasmic reticulum). (G) Quantitative analysis of the distance between ER and mitochondria.*p < 0.05, **p < 0.01, and ****p < 0.001 indicating statistical significance. Each experimental procedure was biologically replicated three times, each yielding similar results.
**Figure S12:** The expression of Ero1L is positively correlated with the degree of senescence in Los patients. (A) Immunohistochemistry for Ero1L in human skin (positive staining appears brown. Control: *n* = 15, Morphea: *n* = 30, scale bar = 20 μm). (B) AOD levels of Ero1L. (C) The correlation analysis of Ero1L with β‐galactosidase. (D) The correlation analysis of Ero1L with mLoSSI.
**Table S1:** Comparison of general materials in two groups.
**Table S2:** The primer sequences.
**Table S3:** Detailed antibody information.
**Table S4:** The primer sequences.
**Table S5:** The top 100 targets in reverse virtual screening.

## Data Availability

The datasets used and analyzed during the current study are available from the corresponding author on reasonable request.
