# Supplementary material for: Targeting Ero1L by Parthenolide Alleviates Cellular Senescence and Fibrosis of Localized Scleroderma by Regulating Mitochondria‐Associated Endoplasmic Reticulum Membranes Stabilization
Source: Aging Cell. 2026 Sep 29;25(10):e70738. doi: 10.1111/acel.70738 (PMC13624378; doi:10.1111/acel.70738)
Supplement: Supplementary file 1 [file ACEL-25-e70738-s001.docx]

**Supplementary Material**

1. **Supplementary Figures and Tables**
   1.1 **Supplementary Figures**

**Fig. S1** The optimal concentration and optimal action time of PTL on HDFs. (A) Determine the optimal concentration of PTL. (B) Determine the optimal action time of PTL. (C-F) Western blot analysis was performed to assess the expression of P21, α SMA and COL1A1 proteins in HDF cells from each group; Semi-quantitative analysis was conducted with normalization to β Actin as the internal control (n = 3).**^＊^***P < 0.5*, **^＊＊^***P < 0.01* indicating statistical significance. Each experimental procedure was biologically replicated three times, each yielding similar results.

**Fig. S2** EdU assay determined the EdU positive rate. A Representative immunofluorescence images of of EdU staining. B Quantification of EdU positive cells (n = 3), six visual fields were selected for counting in each picture. Percent EdU = EdU (+)/Hoechst 33342 (+) × 100. Each experimental procedure was biologically replicated three times, each yielding similar results.

**Fig. S3** GSEA analysis of Ero1L (Control VS TGF β).

**Fig. S4** Overexpression of Ero1L inhibited the anti-fibrotic and anti-senescent effects of PTL. (A-D) Immunofluorescence, western blotting (WB) and qRT-PCR results demonstrated that Ero1L is overexpressed in HDF cells (n=3). (E-J) Western blot analysis was performed to assess the expression of proteins in HDF cells from each group; Semi-quantitative analysis was conducted with normalization to β Actin as the internal control (n=3). **p* < 0.05, ***p* < 0.01, and ****p* < 0.001 indicating statistical significance. Each experimental procedure was biologically replicated three times, each yielding similar results.

**Fig. S5** Overexpression of Ero1L increased mitochondrial calcium, interaction level between IP3R, GRP75 and VDAC1 even with the presence of PTL. (A C) The calcium levels in the cells were measured by flow cytometry and quantification of calcium levels (n=3). (B D E F)Western blot analysis was performed to assess the expression of IP3R, GRP75 and VDAC1 proteins in HDF cells from each group; Semi-quantitative analysis was conducted with normalization to β Actin as the internal control (n = 3). (G) Co-immunoprecipitation (Co-IP) assay showing the interaction between IP3R, GRP75 and VDAC1 (n=3). **p* < 0.05, ***p* < 0.01, and ****p* < 0.001 indicating statistical significance. Each experimental procedure was biologically replicated three times, each yielding similar results.

**Fig. S6** Ero1L knockdown in HDF cells produced anti-senescent and anti-fibrotic effects. (A-C) Western blotting and PCR results showing ERO1L knockdown efficiency in HDF cells (n=3). (D E) Western blotting analysis confirmed successful knockdown of Ero1L in subsequent experiments; Semi-quantitative analysis was conducted with normalization to β Actin as the internal control (n = 3). (F-K) Western blot analysis was performed to assess the expression of COL1A1, α SMA, P21 and P16 proteins in HDF cells from each group; Semi-quantitative analysis was conducted with normalization to β Actin as the internal control (n = 3). (L-N) The expression of TNF α, IL-1β and IL-6 in cell supernatants was tested by ELISA.**p* < 0.05, ***p* < 0.01, and ***p < 0.001 indicating statistical significance. Each experimental procedure was biologically replicated three times, each yielding similar results.

**Fig. S7** Ero1L knockdown helped to maintain MAM homeostasis and alleviate mitochondrial injury in HDF cells. (A-D, I) The mitoSOX red mitochondrial superoxide indicator was used to quantify mitochondrial ROS production. (n=3) (E-H) The mitochondrial membrane potential (MMP) was tested by the JC-1 assay to evaluate the mitochondrial activity. (n=3) (J) Quantification of MMP levels. (K) The quantification of the confocal immunofluorescence results. (L) Laser confocal detection the colocalization of mitochondria and ER. (Scale bar = 5 μm, n=3). (M) TEM was used to monitor ultrastructural changes of the mitochondrion and endoplasmic reticulum ( Scale bar = 500 nm, n=3, The blue arrow represents mitochondria; the red arrow represents endoplasmic reticulum). (N) Quantitative analysis of the distance between ER and mitochondria. (**p < 0.05, **p < 0.01, and ***p < 0.001 indicating statistical significance.*) Each experimental procedure was biologically replicated three times, each yielding similar results.

**Fig. S8** knockdown of Ero1L decreased mitochondrial calcium, interaction level between IP3R, GRP75 and VDAC1. (A-E) The calcium levels in the cells were measured by flow cytometry and quantification of calcium levels (n=3). (F H I J)Western blot analysis was performed to assess the expression of IP3R, GRP75 and VDAC1 proteins in HDF cells from each group; Semi-quantitative analysis was conducted with normalization to β Actin as the internal control (n=3). (G) Co-immunoprecipitation (Co-IP) assay showing the interaction between IP3R, GRP75 and VDAC1. **p < 0.05* and ***p < 0.01* indicating statistical significance. Each experimental procedure was biologically replicated three times, each yielding similar results.

**Fig. S9 HE and Masson trichrome staining.** (A) HE staining. (B) Masson staining.

**Fig. S10** Characteristics of F127@PLGA@PTL. (A) Electron microscopy analysis (Scale bar = 100 nm, n=3). (B) Water solubility of PTL, PLGA@PTL, and PTL plus PLGA. (C-D) Injectability properties of F127@PLGA@PTL. (E) Thermoresponsive properties of F127@PLGA@PTL. (F) SEM images of F127 and F127@PLGA@PTL. (G-H) XRD pattern of PTL and PLGA@PTL. (I-K) Temperature, frequency, and strain rheological curve figure of F127 and F127@PLGA@PTL. (L) Drug release profiles of PTL, PLGA@PTL, and F127@PLGA@PTL. (M) Live/dead cell staining illustrates viable (green) or dead (red) cells. (N) CCK8 assay showing cell viability in different groups and different time periods (n = 6 biological replicates).

**Fig. S11** TPL@PLGA@F127 could regulate calcium ion channel complex and MAM stabilization in vivo. (A-D)Western blot analysis was performed to assess the expression of IP3R, GRP75 and VDAC1 proteins in HDF cells from each group; Semi-quantitative analysis was conducted with normalization to β Actin as the internal control (n=3). (E) Co-immunoprecipitation (Co-IP) assay showing the interaction between IP3R, GRP75 and VDAC1. (F) TEM was used to monitor ultrastructural changes of the mitochondrion and endoplasmic reticulum ( Scale bar = 500 nm, n=3, The red arrow represents mitochondria; the blue arrow represents endoplasmic reticulum). (G) Quantitative analysis of the distance between ER and mitochondria.**p* < 0.05, ***p* < 0.01, and *****p* < 0.001 indicating statistical significance. Each experimental procedure was biologically replicated three times, each yielding similar results.

Fig. S12 The expression of Ero1L is positively correlated with the degree of senescence in Los patients. (A) Immunohistochemistry for Ero1L in human skin (positive staining appears brown. Control: n=15, Morphea: n=30, scale bar = 20 μm). (B) AOD levels of Ero1L. (C) The correlation analysis of Ero1L with β-galactosidase. (D) The correlation analysis of Ero1L with mLoSSI.

1.2 **Supplementary Tables**

Table S1 Comparison of general materials in two groups.

| Groups | n | Age | Sex | |
| --- | --- | --- | --- | --- |
|  |  |  | Male | Female |
| Control | 15 | 31.67±16.51 | 7 | 23 |
| Morphea | 30 | 36.33±14.05 | 4 | 11 |
| t |  | -0.991 |  |  |
| X^2^ |  |  | 0.060 | |
| *P* | >0.05 |  | >0.05 |  |

**Table S2** The primer sequences.

| **Primer** | **Sequence** |
| --- | --- |
| CoL1A1 FP | AAAGATGGACTCAACGGTCTC |
| CoL1A1 RP | CATCGTGAGCCTTCTCTTGAG |
| α SMA FP | CTTGAGAAGAGTTACGAGTTG |
| α SMA RP | GATGCTGTTGTAGGTGGTT |
| P16 FP | CACGGGTCGGGTGAGAGT |
| P16 FP | CCCAACGCACCGAATAGTTAC |
| P21 FP | GCCTGGACTGTTTTCTCTCG |
| P21 RP | ATTCAGCATTGTGGGAGGAG |
| ERO1L FP | GGGCACTGCTCTGAAGATCTTAT |
| ERO1L RP | TGTCCAGAAACAGGCACATATCA |
| IP3R1 FP | GAGAAGGCCGAAGATACCAAGAT |
| IP3R1 RP | TCTCACTGCAAAGCTGGAAATTG |
| Grp75 FP | AAAGACAACATGGCACTTCAGAG |
| Grp75 RP | GAGCACGGGTCAACTTCATATTC |
| VDAC1 FP | CTGGACAGCAGGAAACAGTAACA |
| VDAC1 RP | GACGTTCTTGCCATCCAGAAGAG |
| GAPDH FP | AGGGCTGCTTTTAACTCTGGT |
| GAPDH RP | CCCCACTTGATTTTGGAGGGA |

**Table S3** Detailed antibody information.

| **Antibody** | **Number** | **Antibody dilution** |
| --- | --- | --- |
| Anti-Ero1L | 12007-1-AP | 1:1000 |
| Anti-COL1A1 | 10800-1-AP | 1:1000 |
| Anti-COL1A1 | A1352 | 1:500 |
| Anti-α SMA | 14395-1-AP | 1:5000 |
| Anti-P21 | 82669-2-PR | 1:5000 |
| Anti-P16 | ab51243 | 1:5000 |
| Anti-IP3R1 | ab121664 | 1:500 |
| Anti-GRP75 | 14887-1-AP | 1:10000 |
| Anti-VDAC1 | 10866-1-AP | 1:2000 |

**Table S4** The primer sequences.

| **Primer** | **Sequence** |
| --- | --- |
| Ero1L siRNA FP | GACCAAGCAUGAUGAUUCUTT |
| Ero1L siRNA RP | AGAAUCAUCAUGCUUGGUCTT |
| nontargeting siRNA FP | UUCUCCGAACGUGUCACGUTT |
| nontargeting siRNA RP | ACGUGACACGUUCGGAGAATT |

**Table S5** The top 100 targets in reverse virtual screening.

| **Target name** | **Rank** | **Binding energy** | **Target name** | **Rank** | **Binding energy** |
| --- | --- | --- | --- | --- | --- |
| MYH6 | 1 | -7.887 | RN7SKP2 | 51 | -7.296 |
| CKM | 2 | -7.796 | GGT1 | 52 | -7.295 |
| TNNT2 | 3 | -7.62 | LOC100996455 | 53 | -7.291 |
| TECRL | 4 | -7.616 | RD3L | 54 | -7.283 |
| NRAP | 5 | -7.567 | MESTIT1 | 55 | -7.28 |
| MB | 6 | -7.499 | SLC7A10 | 56 | -7.279 |
| ADIPOQ | 7 | -7.487 | TTYH1 | 57 | -7.277 |
| LMOD2 | 8 | -7.478 | LGALS8-AS1 | 58 | -7.275 |
| SCN7A | 9 | -7.465 | PLIN4 | 59 | -7.275 |
| MYOM2 | 10 | -7.453 | CERS1 | 60 | -7.273 |
| RELN | 11 | -7.449 | MYL12AP1 | 61 | -7.273 |
| NPPA | 12 | -7.448 | RPL22P17 | 62 | -7.269 |
| DAO | 13 | -7.447 | KIF26A | 63 | -7.266 |
| NPY6R | 14 | -7.441 | EXOC3L1 | 64 | -7.262 |
| CCL15-CCL14 | 15 | -7.438 | DCANP1 | 65 | -7.26 |
| LSAMP | 16 | -7.435 | PAPLN | 66 | -7.255 |
| TNNI3 | 17 | -7.422 | SLCO1B1 | 67 | -7.251 |
| PMP2 | 18 | -7.412 | DSG1 | 68 | -7.251 |
| ERO1A | 19 | -7.395 | MYZAP | 69 | -7.248 |
| CRYZP2 | 20 | -7.391 | ZNF663P | 70 | -7.238 |
| RAB3B | 21 | -7.39 | LOC646730 | 71 | -7.234 |
| LOC100996622 | 22 | -7.387 | TRGV10 | 72 | -7.233 |
| LOC101928177 | 23 | -7.386 | PTPRH | 73 | -7.231 |
| SERPIND1 | 24 | -7.382 | PTPN5 | 74 | -7.224 |
| LPPR4 | 25 | -7.37 | HNRNPA1P33 | 75 | -7.223 |
| SELP | 26 | -7.369 | PRRG3 | 76 | -7.223 |
| RUNDC3A | 27 | -7.369 | ATP10B | 77 | -7.222 |
| UNC79 | 28 | -7.368 | CRABP1 | 78 | -7.22 |
| MIR579 | 29 | -7.365 | LOC100421901 | 79 | -7.22 |
| GLYATL2 | 30 | -7.363 | LOC100505902 | 80 | -7.215 |
| USH2A | 31 | -7.354 | SPIB | 81 | -7.21 |
| PI16 | 32 | -7.353 | MIR432 | 82 | -7.205 |
| RPL32P13 | 33 | -7.352 | SLCO6A1 | 83 | -7.204 |
| ATP8A2 | 34 | -7.351 | TCF15 | 84 | -7.203 |
| MIR581 | 35 | -7.348 | MAG | 85 | -7.203 |
| LOC101927366 | 36 | -7.346 | VSTM5 | 86 | -7.202 |
| SEL1L2 | 37 | -7.346 | EBF2 | 87 | -7.201 |
| C10orf82 | 38 | -7.342 | SLC7A14 | 88 | -7.201 |
| PITX2 | 39 | -7.342 | HSD11B2 | 89 | -7.2 |
| SEMA3G | 40 | -7.335 | TRD-GTC10-1 | 90 | -7.198 |
| LOC101928558 | 41 | -7.322 | SCARA5 | 91 | -7.197 |
| CFD | 42 | -7.321 | LHX6 | 92 | -7.195 |
| HSPB3 | 43 | -7.321 | LOC100422432 | 93 | -7.195 |
| ECEL1 | 44 | -7.316 | VWFP1 | 94 | -7.195 |
| CHAD | 45 | -7.309 | SLC26A5 | 95 | -7.193 |
| LCN6 | 46 | -7.303 | HRAT56 | 96 | -7.192 |
| LOC102724479 | 47 | -7.298 | FOXI2 | 97 | -7.191 |
| LHFPL3-AS1 | 48 | -7.298 | TRAJ33 | 98 | -7.189 |
| C14orf39 | 49 | -7.298 | TCEA1P3 | 99 | -7.187 |
| LOC101927174 | 50 | -7.297 | GRIN2B | 100 | -7.187 |
